# Evaluation of commercial immunodiffusion reagents for detecting serum anti-*Paracoccidioides* antibodies

**DOI:** 10.1590/0037-8682-0094-2024

**Published:** 2024-05-27

**Authors:** Regielly Caroline Raimundo Cognialli, Diego H. Caceres, Flávio de Queiroz Telles

**Affiliations:** 1 Universidade Federal do Paraná, Hospital de Clínicas, Curitiba, PR, Brasil.; 2 Center of Expertise in Mycology Radboudumc/CWZ. Nijmegen, the Netherlands.; 3 Universidad del Rosario, Studies in Translational Microbiology and Faculty of Medical Sciences, Emerging Diseases Research Group. Bogota, Colombia.; 4 IMMY. Norma, OK, United States of America.; 5 Universidade Federal do Paraná, Hospital de Clínicas, Departamento de Saúde Coletiva, Curitiba, PR, Brasil.

**Keywords:** Double immunodiffusion, Paracoccidioides, Antibodies

## Abstract

**Background:**

Accurate diagnosis of paracoccidioidomycosis is crucial for improving patient outcomes. *Paracoccidioides* antibody detection by double immunodiffusion (DID) is a convenient diagnostic tool, but testing performance can vary based on certain factors.

**Methods:**

We assessed DID performance using a commercially prepared *Paracoccidioides* reagents (IMMY, USA), involving 40 serum specimens, including 20 from patients with proven paracoccidioidomycosis and 20 from patients without the disease. The DID test demonstrated a sensitivity of 90% (95% CI=68%-99%) and a specificity of 100% (95% CI=83%-100%).

**Conclusions:**

Our findings suggest that DID using commercial reagents may provide a feasible tool with satisfactory testing performance for anti-*Paracoccidioides* antibody detection.

Paracoccidioidomycosis (PCM) is an endemic systemic mycosis in Latin America, with most cases (~80%) reported in Brazil^1^ ^-^ ^3^. PCM is caused by *Paracoccidioides* spp., a thermally dimorphic fungus that encompasses *P. brasiliensis* complex and *P. lutzii* ^1^ ^,^ ^2^. Phylogenetic studies have identified four cryptic species within the *P. brasiliensis* complex: *P. brasiliensis sensu stricto* (S1)*, P. americana* (PS2)*, P. restrepiensis* (PS3)*,* and *P. venezuelensis* (PS4)^1^. PCM most frequently affects males from rural regions and is considered an occupational disease^1^ ^,^ ^4^. Infection occurs through the inhalation of propagules of the mycelial phase and microconidia^2^. Only 1-2% of infected individuals develop symptoms, and the clinical forms of PCM can be categorized into acute/subacute and chronic^2^ ^-^ ^4^. The global burden of PCM is estimated at 4,000 cases per year, and its annual incidence in Brazil ranges from 0.71 to 40 cases per 100,000 inhabitants, with a 6.1%-7.6% mortality rate^4^ ^-^ ^6^. PCM can affect different organs and has a broad spectrum of clinical manifestations^3^ ^,^ ^4^. Pulmonary PCM is frequently misdiagnosed as tuberculosis, which delays antifungal treatment and increases morbidity and mortality^4^ ^,^ ^5^ ^,^ ^7^. Therefore, accurate laboratory diagnosis is fundamental for differential diagnosis.

The standard method for laboratory diagnosis of PCM involves either direct visualization of yeast cells in clinical specimens or fungal isolation in culture^8^. However, the sensitivity of these methods depends on the specimen type and the operator's expertise, and the slow growth of the fungus often leads to diagnostic delays^1^ ^,^ ^2^ ^,^ ^5^ ^,^ ^8^ ^,^ ^9^. Immunodiagnostic assays allow for rapid diagnosis and prompt antifungal therapy^2^ ^-^ ^4^. Antibody detection can be performed using different methods, but diffusion methods such as double immunodiffusion (DID) and counterimmunoelectrophoresis are the most used^3^ ^,^ ^8^ ^-^ ^11^. DID is the test of choice for antibody detection in PCM diagnosis and is widely used in countries where PCM is endemic^2^ ^,^ ^10^ ^-^ ^12^. However, limited access to commercial kits significantly hampers the implementation of DID^5^ ^,^ ^8^. Furthermore, the antigen is prepared *in-house*, which can lead to a lack of standardization in antigen preparation with respect to the strain, culture media, growth conditions, and DID gel plates^2^ ^,^ ^5^. These factors contribute to variations in the sensitivity and specificity of the antibody detection assays^1^ ^,^ ^4^ ^,^ ^12^. This study aimed to evaluate commercial DID reagents for detecting anti-*Paracoccidioides* antibodies (Ab) in serum specimens. 

This was a retrospective experimental study. We included a total of 40 serum specimens, which had been stored at -20°C since 2011. The specimens were divided into three groups. Group 1 consisted of 20 sera from patients with PCM, including six from patients with a culture-proven diagnosis and 14 from patients with a positive microscopic examination. We evaluated patients with PCM regardless of sex, clinical form, or age. Furthermore, Group 1 was subdivided into patients with a new diagnosis (n=8) and those undergoing treatment follow-up (n=12), with the latter tested from six months to 12 months post-treatment initiation. Group 2 comprised ten serum samples from non-symptomatic volunteers. Group 3 included ten serum specimens from patients with proven diagnosis of mycosis other than PCM: four with sporotrichosis, two with disseminated histoplasmosis, two with invasive fusariosis, and two with invasive aspergillosis (Figure 1). This study received approval from the HC-UFPR Research Ethics Committee under registration CAAE 73792023.6.0000.0096.

**FIGURE 1:**
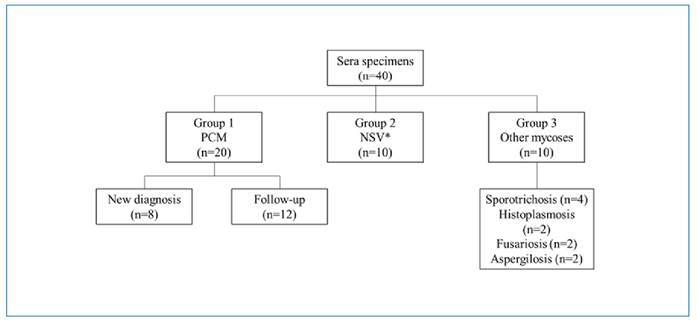
Flow chart of study design for evaluating commercial immunodiffusion reagents for the detection of anti-*Paracoccidioides* antibodies in sera. **NSV:** non-symptomatic volunteers.

DID was performed using the commercial *Paracoccidioides* antigen (Ag), control sera, and Cleargel^TM^ immunodiffusion plates produced by IMMY (Norman, OK, USA). Specimens were stored at -20°C; antigen and DID plates were stored at 2-8°C until testing. 

Serum specimens were tested both undiluted and diluted with saline solution (ranging from 1:2 to 1:16). The wells of the DID plates were filled with undiluted serum, diluted serum (1:2, 1:4, 1:8, and 1:16) and control serum. A positive serum control was included in all reactions. Thirty minutes after adding the specimens, the central well was filled with *Paracoccidioides* Ag. The DID plates were then incubated at room temperature for 24 and 48 hours. Results were visually assessed under a high-intensity light against a dark background (Figure 2).

**FIGURE 2:**
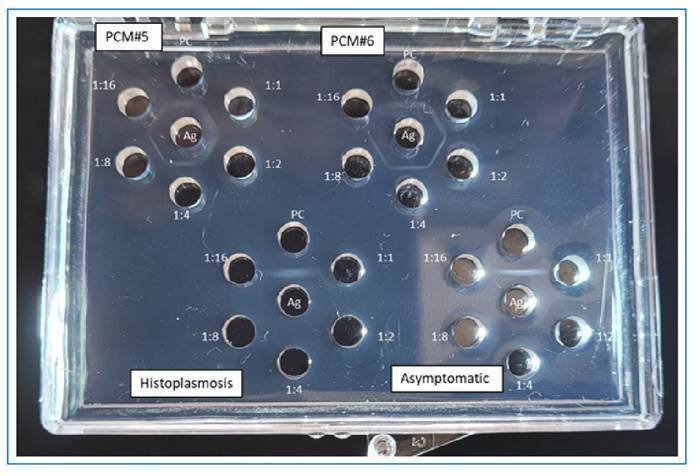
Example of DID results for the detection of anti-*Paracoccidioides* antibodies in serum specimens. Ag - *Paracoccidioides* antigen in the center well; PC: positive control; 1:1, undiluted serum; 1:2-1:16 dilution. For PCM#5 and PCM#6 sera from patients with proven paracoccidioidomycosis, the positive results were 1:16 and 1:8, respectively. **Histoplasmosis:** Sera from patients with proven histoplasmosis, negative results (only precipitin band on positive control). **Asymptomatic:** Sera from an asymptomatic volunteer, negative result (only precipitin band on the positive control).

DID demonstrated a sensitivity of 90% (95% confidence interval [CI], 68%-99%). Two false-negative results were recorded: one from a patient with severe immunosuppression and another from a patient with central nervous system PCM. Patients with PCM displayed antibody titers ranging from undiluted to 1:32. Newly diagnosed patients showed antibody titers from 1:8 to 1:32, while patients in treatment follow-up presented titers from undiluted to 1:16. No false-positive results were observed, specificity at 100% (95% CI=83%-100%) (Table 1).

**TABLE 1:** Analytical performance of DID for the detection of anti-*Paracoccidioides* antibodies.

		**PCM diagnosis**	
		+	-
**IMMY DID**	+	18	0
	-	2	20
		**% (95% CI)**	
Sensitivity		90 (68-99)	
Specificity		100 (83-100)	
Positive predictive value		100 (81-100)	
Negative predictive value		91 (73-97)	
Accuracy		88 (83-99)	

Legend: −: Negative; +: Positive; 95% CI: 95% confidence interval.

Assays based on agar diffusion are characterized by variable sensitivities, ranging from 75% to 95%^8^ ^-^ ^10^. Different factors could influence testing performance, including the diversity of *Paracoccidioides* isolates used for antigen production, the concentration of the antigen, the agar used for diffusion, low-avidity IgG2 antibodies directed against carbohydrate epitopes, the clinical form, and the patient’s immune status^5^ ^,^ ^11^ ^-^ ^13^. Nonetheless, we observed high performance from the DID reagents evaluated, establishing this commercial kit as a suitable option for diagnosing PCM. False-negative results have been noted in patients with immunosuppression and meningitis; these conditions are associated with low antibody concentrations, which may compromise the effectiveness of antibody detection assays in these patients^4^ ^,^ ^5^ ^,^ ^13^. No false-positive results were recorded. Cross-reactivity with other fungal infections has been reported, primarily in sera from patients with histoplasmosis, due to the similarity of glycoproteins antigens^11^ ^,^ ^14^ *.* However, more studies are needed to evaluate cross-reactivity.

This commercial *Paracoccidioides* ID kit had previously been evaluated using counterimmunoelectrophoresis and a DID on *in-house* agar plates, achieving a sensitivity of 75% and a specificity of 100%^10^. The higher sensitivity observed in this study can be attributed to the use of Cleargel plates.

The DID reagents produced by IMMY utilize *Paracoccidioides* antigens extracted from the culture filtrate during the mycelial phase of *P. brasiliensis* (ATCC PB339). This strain includes the glycoprotein gp43, among other antigens. The serum specimens evaluated in this study were sourced from patients with PCM residing in the Brazilian state of Paraná, where previous research indicates that most cases are caused by the *P. brasiliensis* complex^15^. Of the 20 patients evaluated, six were proven by culture. One of these six cultures was identified as belonging to the *Paracoccidioides brasiliensis* complex by sequencing the internal transcribed spacer (ITS) region of the ribosomal DNA. Therefore, the main limitation of this study was the non-inclusion of sera from patients with infections caused by *P. lutzii*.

Immunodiagnostic tests for PCM are crucial as they enable rapid diagnosis and are useful for monitoring treatment. Currently, DID is considered the gold standard for antibody detection; however, significant interlaboratory variations persist, largely due to the use of *in-house* produced antigens^5^ *.* IMMY's *Paracoccidioides* antigen enables standardization, and Cleargel^TM^ plates facilitate the easy visualization of precipitin bands without the need for staining or additional reagents. Employing commercial reagents for DID is straightforward and could provide a suitable alternative for multicenter testing of anti-*Paracoccidioides* antibodies.

## References

[1] Hahn RC, Hagen F, Mendes RP, Burger E, Nery AF, Siqueira NP (2022). Paracoccidioidomycosis: Current Status and Future Trends. Clin Microbiol Rev.

[2] Peçanha PM, Peçanha-Pietrobom PM, Grão-Velloso TR, Rosa M, Falqueto A, Gonçalves SS (2022). Paracoccidioidomycosis: What We Know and What Is New in Epidemiology, Diagnosis, and Treatment. J Fungi.

[3] Shikanai-Yasuda MA, Mendes RP, Colombo AL, Queiroz-Telles F, Kono ASG, Paniago AMM (2017). Brazilian guidelines for the clinical management of paracoccidioidomycosis. Rev Soc Bras Med Trop.

[4] Mendes RP, Cavalcante RS, Marques SA, Marques MEA, Venturini J, Sylvestre TF (2017). Paracoccidioidomycosis: Current Perspectives from Brazil. Open Microbiol J.

[5] Griffiths J, Lopes Colombo A, Denning DW (2019). The case for paracoccidioidomycosis to be accepted as a neglected tropical (fungal) disease. PLoS Negl Trop Dis.

[6] Martinez R (2017). New Trends in Paracoccidioidomycosis Epidemiology. J Fungi.

[7] Queiroz-Telles FV, Peçanha Pietrobom PM, Rosa M, Baptista RM, Peçanha PM (2020). New Insights on Pulmonary Paracoccidioidomycosis. Semin Respir Crit Care Med.

[8] Peçanha-Pietrobom PM, Tirado-Sánchez A, Gonçalves SS, Bonifaz A, Colombo AL (2023). Diagnosis and Treatment of Pulmonary Coccidioidomycosis and Paracoccidioidomycosis. J Fungi.

[9] Moreto TC, Marques ME, de Oliveira ML, Moris DV, de Carvalho LR, Mendes RP (2011). Accuracy of routine diagnostic tests used in paracoccidioidomycosis patients at a university hospital. Trans R Soc Trop Med Hyg.

[10] Cocio TA, Martinez R (2021). Serological diagnosis of paracoccidioidomycosis using a Paracoccidioides spp. comercial antigen and the counterimmunoelectrophoresis method. Braz J Infect Dis.

[11] Maifrede SB, Kruschewsky WLL, Patrício SA, Falqueto A, Peçanha PM, Malaquias LCC (2021). Screening paracoccidioidomycosis by double immunodiffusion test in a referred diagnostic center in Brazilian southeastern: an accessible tool. Infection.

[12] Vidal MS, Del Negro GM, Vicentini AP, Svidzinski TI, Mendes-Giannini MJ, Almeida AM (2014). Serological diagnosis of paracoccidioidomycosis: high rate of inter-laboratorial variability among medical mycology reference centers. PLoS Negl Trop Dis.

[13] Neves AR, Mamoni RL, Rossi CL, de Camargo ZP, Blotta MH (2003). Negative immunodiffusion test results obtained with sera of paracoccidioidomycosis patients may be related to low-avidity immunoglobulin G2 antibodies directed against carbohydrate epitopes. Clin Diagn Lab Immunol.

[14] Alvarado P, Pérez-Rojas Y, Zambrano EA, Gonzatti MI, Roschman-González A (2020). Improved serodiagnosis of histoplasmosis by use of deglycosylated extracellular released antigens of Histoplasma capsulatum. J Microbiol Methods.

[15] Rodrigues AM, Hagen F, Puccia R, Hahn RC, de Camargo ZP (2023). Paracoccidioides and Paracoccidioidomycosis in the 21st Century. Mycopathologia.

